# Antioxidant and Cytoprotective Effects of Herbal Extract-Enriched Artificial Saliva: In-Vitro Biocompatibility and Cell Culture Studies

**DOI:** 10.3290/j.ohpd.c_2552

**Published:** 2026-03-11

**Authors:** Hülya Erten Can, Asli Silkü Oflioğlu, Nasibe Aycan Yilmaz, Kadriye Merve Altikat, Rukiye Yavaşer Boncooğlu, Gamze Tuna, Göksu Derinsu

**Affiliations:** a Hülya Erten Can Professor, Dokuz Eylul University, Department of Restorative Dentistry, İzmir, Türkiye. Conception of the study idea, creation of experimental material, read and approved the final manuscript.; b Aslı Silkü Oflioğlu Research Assistant, Dokuz Eylul University, Department of Restorative Dentistry, İzmir, Türkiye. Preparation of the samples, author of the manuscript. Submission and revision of the manuscript.; c Nasibe Aycan Yilmaz Associate Professor, Dokuz Eylul University, Department of Restorative Dentistry, İzmir, Türkiye. Study idea.; d Kadriye Merve Altikat Assistant Professor, Istinye University, Oral and Dental Health Program, Türkiye. Data analysis and interpretation of the cell culture study.; e Rukiye Yavaşer Boncooğlu Assistant Professor, Aydın Adnan Menderes University, Faculty of Science, Chemistry Department, Aydın, Türkiye. Studying the antioxidant values of samples.; f Gamze Tuna Associate Professor, Dokuz Eylul University, Institute of Health Sciences, Department of Molecular Medicine, Izmir, Türkiye. Laboratory facilities and laboratory training.; g Göksu Derinsu Research Assistant, Izmir Katip Çelebi University, Department of Oral and Maxillofacial Radiology, Izmir, Türkiye. Preparation of the samples.

**Keywords:** antioxidant activity, artificial saliva, cytotoxicity, dry mouth, plant extracts.

## Abstract

**Purpose:**

The aim of this in-vitro exploratory study was to develop and evaluate a biocompatible and cost-effective artificial saliva formulation using locally available natural resources for the supportive management of xerostomia.

**Materials and Methods:**

Twenty-four experimental artificial saliva formulations were prepared using two base matrices (mucin-based and carboxymethyl-cellulose–based) at two viscosity levels. Five plant extracts — Olea europaea (olive leaf), Cynara cardunculus (artichoke leaf), Equisetum arvense (horsetail), Hypericum perforatum (St. John’s Wort), Origanum vulgare (thyme) — were incorporated together with extract-free control formulations. The formulations were evaluated in-vitro in terms of viscosity, cytocompatibility on gingival fibroblasts, antioxidant capacity, and antimicrobial activity.

**Results:**

The formulations developed demonstrated viscosities within the physiological range of natural saliva. Cytotoxicity testing using gingival fibroblasts showed no detectable cytotoxic effects. Extract-containing formulations exhibited statistically significantly higher antioxidant activity compared with controls (p < 0.05). Antimicrobial activity assessed by disk diffusion testing revealed no measurable inhibition zones under the applied experimental conditions.

**Conclusion:**

The developed artificial saliva formulations demonstrated favorable physicochemical properties, good cytocompatibility, and enhanced antioxidant activity. These findings suggest that the formulation may represent a promising biomimetic candidate for further investigation in the supportive management of xerostomia.

Saliva is a complex biological fluid that plays essential roles in mastication, swallowing, speech, and the protection of both hard and soft oral tissues, including teeth, tongue, and mucosa.^12^ Owing to its composition rich in electrolytes, enzymes, and proteins, saliva is critical for oral lubrication, antimicrobial defense, and mucosal integrity; therefore, any reduction in salivary flow can have profound consequences for oral health and quality of life.

Xerostomia, defined as insufficient salivary secretion, may arise from salivary gland dysfunction, ductal obstruction, systemic conditions such as diabetes mellitus and menopause, or as a side effect of commonly prescribed medications, including antihypertensive, antiallergic, and antidepressant agents.^29,33,36^ The clinical consequences of salivary hypofunction include oral discomfort, mucosal ulceration, fungal infections, dental caries, and periodontal disease, often accompanied by impaired speech and reduced tolerance to removable prostheses.^11,15,28^

Management of xerostomia primarily targets underlying reversible causes; however, in chronic or irreversible conditions – such as after head and neck radiotherapy – salivary gland dysfunction is permanent, making artificial saliva substitutes an essential component of supportive care.^18,37^

Artificial saliva formulations are designed to compensate for reduced or absent salivary secretion by reproducing the lubricating, protective, and buffering functions of natural saliva.^30^ Over the past few decades, numerous artificial saliva formulations have been developed to improve oral comfort by mimicking the physicochemical properties of natural saliva.^14,35^ Most existing artificial saliva formulations primarily aim to restore lubrication and electrolyte balance, often relying on viscosity-modifying agents such as carboxymethyl cellulose or mucin.^1^ Mucin is a high-molecular-weight glycoprotein that constitutes a key structural component of natural saliva and plays a central role in lubrication, mucosal adhesion, and salivary film formation.^16^ Due to its physiological relevance, mucin has been widely employed in biomimetic saliva substitutes to reproduce the viscoelastic and boundary-lubricating behavior of human saliva.^38^ In contrast, CMC (carboxymethyl cellulose) is a semi-synthetic, water-soluble polysaccharide frequently used in commercial artificial saliva products because of its chemical stability, ease of formulation, and cost-effectiveness.^10^ Although CMC-based formulations can effectively improve oral lubrication, they lack the glycoprotein-mediated biological functions inherent to natural saliva.^35^ Consequently, both mucin- and CMC-based matrices are commonly investigated to compare biomimetic and polymer-based approaches under controlled conditions. Despite these advances, many existing formulations exhibit limited bioactive characterization, and parameters such as antioxidant capacity, cytocompatibility, and long-term stability have yet to be comprehensively evaluated.^13,35^

Human salivary properties relevant to lubrication and protein-mediated colloidal behavior have been well characterized,^11^ and recent experimental work by Zaheer et al^39^ provided important benchmarks for defining physicochemical targets of saliva substitutes under dietary challenges. Building on this framework, recent research has explored the incorporation of antioxidant and antimicrobial agents, bioactive plant extracts, and nanoparticles to enhance the functional performance of artificial saliva formulations.^30,32^ In parallel, evidence from the food and pharmaceutical industries has highlighted the antimicrobial potential of natural products such as cinnamon, mint, tea tree, hibiscus, licorice root, and chitosan, which can be successfully incorporated into aqueous formulations.^23^

However, relatively few studies have focused on locally sourced plant extracts with documented bioactive potential in the context of artificial saliva. The present study was therefore designed as an exploratory in-vitro investigation to examine whether an artificial saliva formulation enriched with selected locally sourced plant extracts could maintain cytocompatibility while exhibiting antioxidant activity under controlled laboratory conditions.

The Aegean region of Turkey (Türkiye) possesses rich botanical diversity, offering numerous plant species with established bioactive potential.^25^ The selection of plant extracts and additives was guided by a rational formulation strategy based on complementary biological functions rather than arbitrary inclusion. *Olea europaea* (olive leaf), *Cynara cardunculus* (artichoke leaf), *Equisetum arvense* (horsetail), *Hypericum perforatum* (St. John’s wort), *Origanum vulgare* (thyme) were selected for their widely reported antioxidant and anti-inflammatory properties relevant to oxidative stress and mucosal vulnerability.^2,6,8^ Such multifunctional formulations combining bioactive plant extracts with biopolymers and natural products have been increasingly explored to achieve complementary and potentially synergistic effects in oral and biomedical applications.^26^ In contrast to conventional artificial saliva formulations that primarily focus on lubrication, the present formulation integrates bioactive plant extracts and biopolymers and is systematically evaluated for antioxidant activity, cytocompatibility, and stability. This approach addresses key limitations of existing formulations.

To examine the influence of formulation rheology on physicochemical and biological performance, two commonly used bioadhesive matrices – mucin and CMC – were employed as base components. Each base formulation was prepared at low and high viscosity to reflect the physiological range of natural saliva and to evaluate potential viscosity-dependent effects on cytocompatibility and antioxidant behavior. In total, 24 experimental formulations were generated by combining two base matrices, two viscosities, five plant-extract–enriched formulations, and corresponding extract-free controls, and were evaluated in-vitro in terms of viscosity and cytocompatibility, as well as antioxidant and antimicrobial activity.

## MATERIALS AND METHODS

### Preparation of Artificial Saliva 

For the pre-production stage of artificial saliva preparation, 1000 ml of prototype was prepared by adding chemicals close to the natural saliva content in appropriate proportions. Accordingly, 0.33 g KH_2_PO_4_, 0.34 g Na_2_HPO_4_, 1.27 g KCl, 0.16 g NaSCN, 0.58 g NaCl, 0.17 g CaCl_2_, 0.16 g NH_4_Cl, 0.2 g urea, 0.03 g glucose, 0.002 g ascorbic acid, and 10 g sodium carboxymethyl cellulose (CMC) were weighed and dissolved in 1000 ml pure water. The pH of the solution was adjusted to 7.0.

In addition to the prepared mixture, a separate preparation was produced using mucin from porcine stomach (Type 2, Sigma-Aldrich; St. Louis, MO, USA), a high-molecular-weight glycoprotein naturally present in human saliva and responsible for lubrication and mucosal adhesion, instead of sodium CMC as a bioadhesive agent.^16^ Both mixtures were tested within the scope of the study.

Plant-derived extracts were obtained as commercially available liquid preparations from a single manufacturer (Kalextract; Balıkesir, Turkey). According to the supplier, all extracts were produced using an ethanol-water solvent system, which is commonly employed for efficient extraction of phenolic and flavonoid compounds.^9^ The extracts included *Olea europaea* (olive leaf), *Cynara cardunculus* (artichoke leaf), *Equisetum arvense* (horsetail), *Hypericum perforatum* (St. John’s Wort), *Origanum vulgare* (thyme).

Each extract was incorporated into the base formulations at a fixed volume of 0.5 ml per sample. Extract-free control formulations were prepared in parallel for each base matrix and viscosity condition. Chemical pre-standardization of individual extracts was not performed, as the primary objective was to assess their functional performance within the final formulation rather than isolated phytochemical profiles. All formulations were sterilized using UV-C irradiation (254 nm, 15 min) under aseptic conditions prior to in-vitro experiments.^20^

### Solution Density and Viscosity Measurements

Solution density was measured using a calibrated glass pycnometer at 25 ± 1°C. The empty pycnometer was weighed, filled with the test solution, and reweighed. Measurements were performed in triplicate, and density was calculated by dividing the net mass of the solution by the calibrated pycnometer volume.

**Fig 1:**
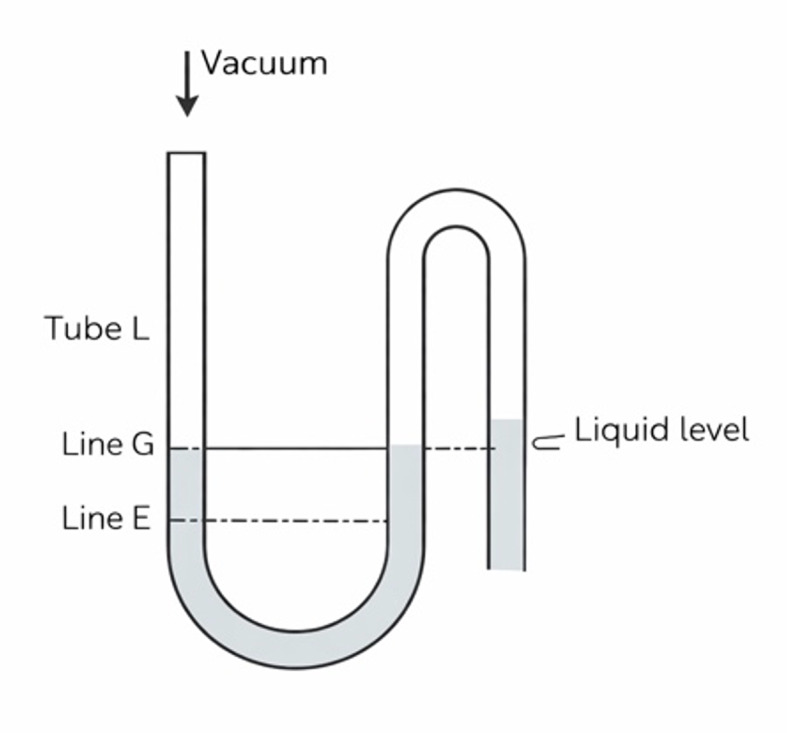
Schematic representation of the U-tube capilary viscometer used for viscosity measurements.

Viscosity measurements were conducted using a U-tube capillary viscometer at 25 ± 1°C. The solution was introduced into tube L above line G, and vacuum was applied until the liquid level reached approximately 5 mm above line E (Fig 1). After release of the vacuum, the flow time of the meniscus between lines E and F was recorded. All measurements were performed in triplicate.

Viscosity was calculated as relative viscosity with reference to distilled water measured under identical conditions, using the ratio of flow times and densities:

η_c_ / η_w_ = (ρ_c_ × t_c_) / (ρ_w_ × t_w_)

where η stands for viscosity, ρ for density, and t for flow time. The subscripts c and w denote the test solution and distilled water, respectively.

This approach was intentionally selected to enable comparative assessment of rheological behavior between formulations under standardized conditions, rather than absolute viscosity determination. Accordingly, direct numerical comparison with absolute viscosity values reported in the literature is limited.

### Stability / Environmental Stress Testing 

Stability testing was performed as exploratory environmental stress screening rather than formal shelf-life or regulatory stability testing. Artificial saliva samples were incubated in controlled environmental chambers at temperatures ranging from 0°C to 99.9°C and relative humidity between 10% and 95%.

Following incubation, samples were evaluated for macroscopic changes in appearance, odor, pH, and density.

These conditions were applied to qualitatively assess formulation robustness under extreme environmental stress and were not intended to simulate physiological storage or establish expiration criteria.

### Evaluation of the Toxicity of Artificial Saliva Using the Gingival Fibroblast Survival Method 

The cytotoxicity of sterilized artificial saliva samples from 24 experimental groups was evaluated using gingival fibroblast cells. Primary human gingival fibroblasts were obtained from a commercially available cryopreserved cell stock (Sigma-Aldrich) and cultured according to the supplier’s instructions. No human or animal tissue was collected specifically for this study.

#### Thawing procedures for gingival cell suspensions

Cells cryopreserved in 1 ml of freezing medium consisting of 95% culture medium and 5% dimethyl sulfoxide (DMSO) were used per cryotube (1.8 ml). Cryotubes were removed from -80°C storage and thawed in a 37°C water bath for approximately 1 min. To remove DMSO, 5 ml of DMSO-free culture medium was added, followed by centrifugation at 1500 rpm for 5 min. The supernatant was discarded, 1 ml of fresh medium was added, and cells were gently resuspended by pipetting. Cell viability and density were assessed using Trypan blue staining (1:1), and cells were seeded into 25 cm² culture flasks according to the determined cell count.

#### Cell passaging 

Culture medium was removed, and cells were washed with phosphate-buffered saline (PBS). Trypsin/EDTA solution was added and incubated for 3 minutes. After neutralization with fresh medium, cells were centrifuged and counted using a hemocytometer. Cells were seeded into 25 cm² flasks containing 5 ml of medium at a density of 0.5 × 10⁶ cells per flask. Cell confluence was monitored at 48- and 72h intervals using an inverted microscope and documented photographically. Cells were expanded up to the fourth passage and used for subsequent experiments.

**Fig 2:**
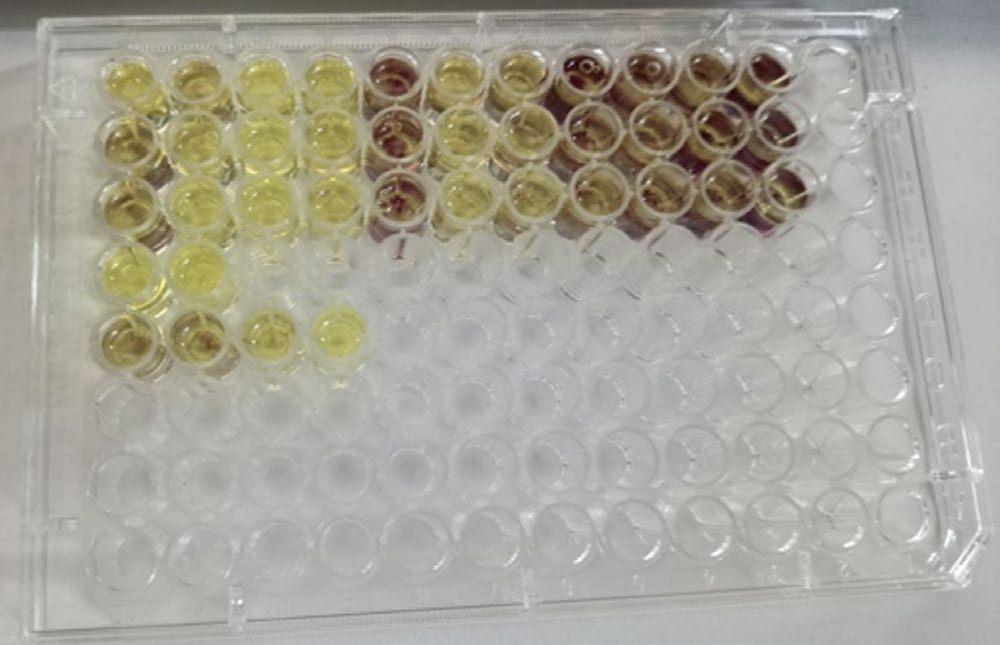
Representative image of the MTT assay performed on gingival fibroblasts after 24 h exposure to different dilutions of artificial saliva formulations. Color change in the wells indicates metabolically active cells and was used to determine the appropriate dilution for subsequent experiments.

**Fig 3:**
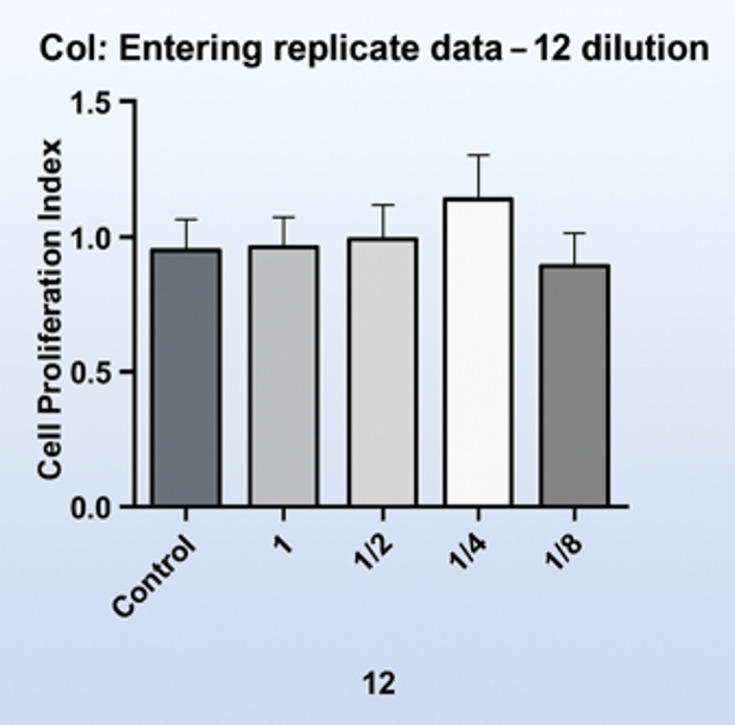

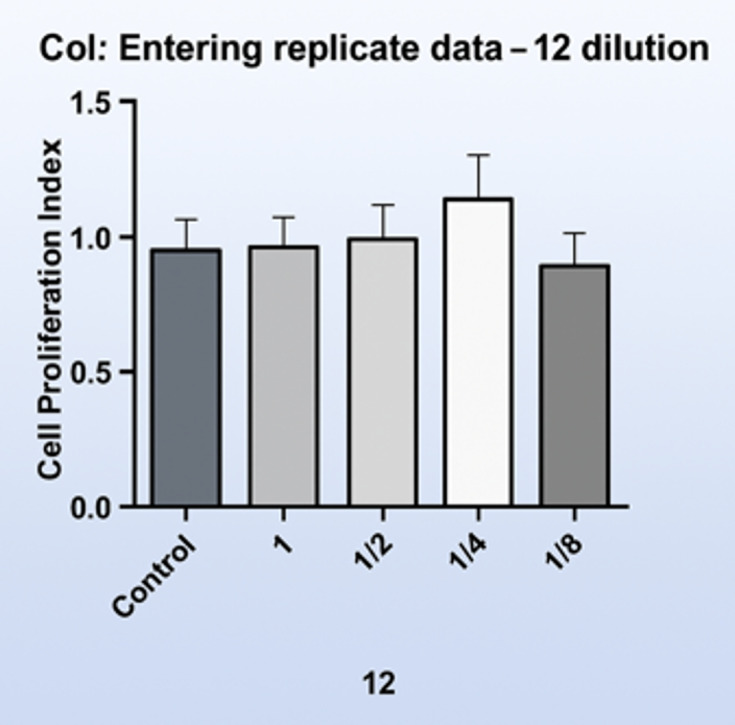
Comparison of cell proliferation index at different dilution ratios (1:2, 1:4, 1:8, and 1:12) in gingival fibroblasts exposed to artificial saliva formulations. Data represent mean ± standard deviation (n = 3). The dilution ratio showing minimal cytotoxicity and optimal cell viability was selected for subsequent experiments.

**Fig 4:**
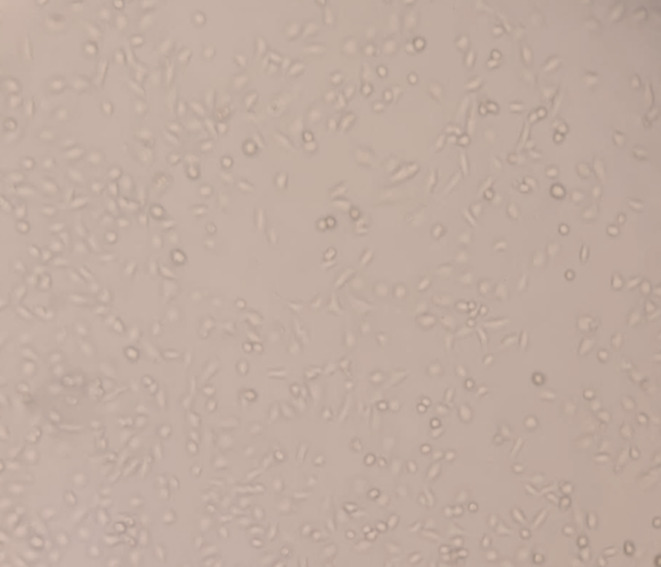

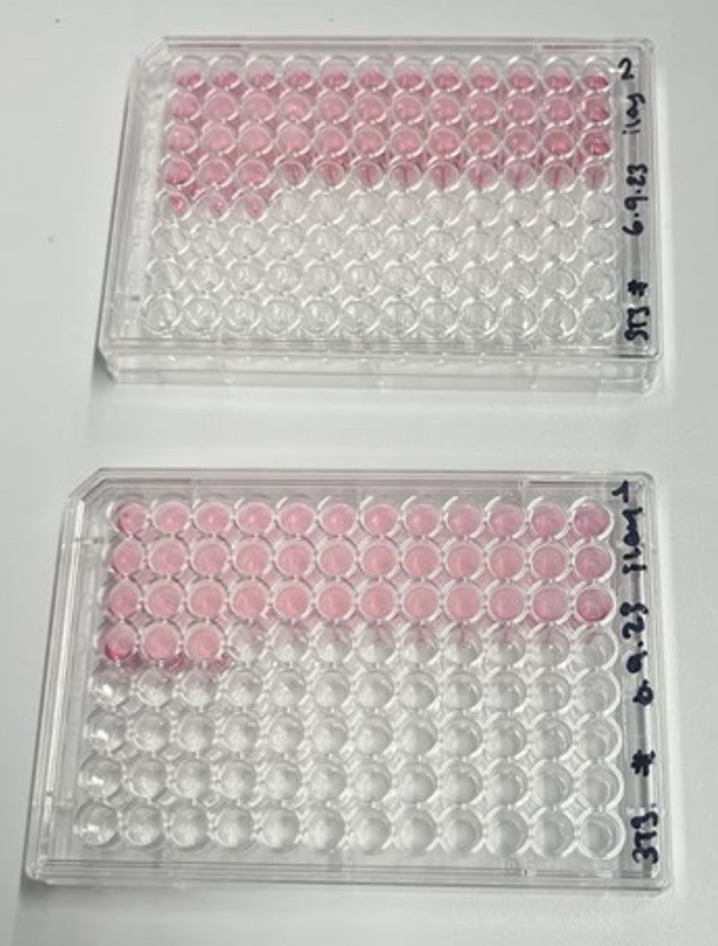
Representative phase-contrast micrographs of gingival fibroblast morphology in the negative control group prior to MTT application. (a) Phase-contrast micrograph showing normal spindle-shaped fibroblast morphology. (b) Representative image of the 96-well plate used in the MTT assay demonstrating colorimetric changes corresponding to metabolically active cells.

**Fig 5:**
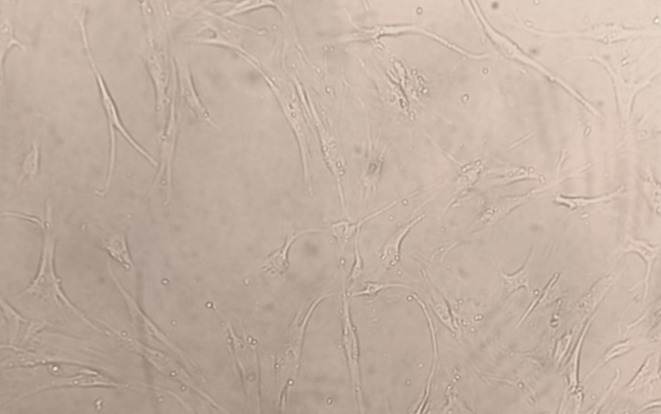
Representative phase-contrast micro-graph of gingival fibroblasts in the negative control group following MTT application. Preserved cell morphology and adherence confirm the absence of morphological damage induced by the assay procedure itself (magnification 200X).

For MTT assays, cells were seeded into 96-well plates in standard culture medium. Prior to analysis, culture medium was removed and confluence was confirmed. MTT reagent was applied at a final concentration of 0.5 mg/ml using a dilution ratio of 1:20 relative to the test formulations, followed by overnight incubation under standard culture conditions (Figs 2 and 3). Following optimization of the dilution factor, all formulations were evaluated after seeding 1 × 10^4^ cells per well and overnight incubation at a final formulation dilution of 1:4. Untreated cells cultured in standard growth medium served as negative controls, while extract-free artificial saliva formulations served as formulation controls (Figs 4 and 5).

### Evaluation of the Antioxidant Activity of Artificial Saliva (DPPH Method)

The antioxidant activity of the artificial saliva preparations was evaluated using the DPPH radical scavenging assay. Antioxidants are known to contribute to cellular protection and repair, and their role in oral health has been widely emphasized in the context of periodontal healing, mucosal lesions, and soft tissue damage. Various in-vitro test systems are available for the identification of antioxidant activity, among which the DPPH radical scavenging assay is one of the most commonly used methods.^5,21,24,27^ The reduction of the DPPH radical, indicated by a color change from purple to yellow, reflects the antioxidant capacity of the tested samples.

#### DPPH radical scavenging activity assay

**Fig 6:**
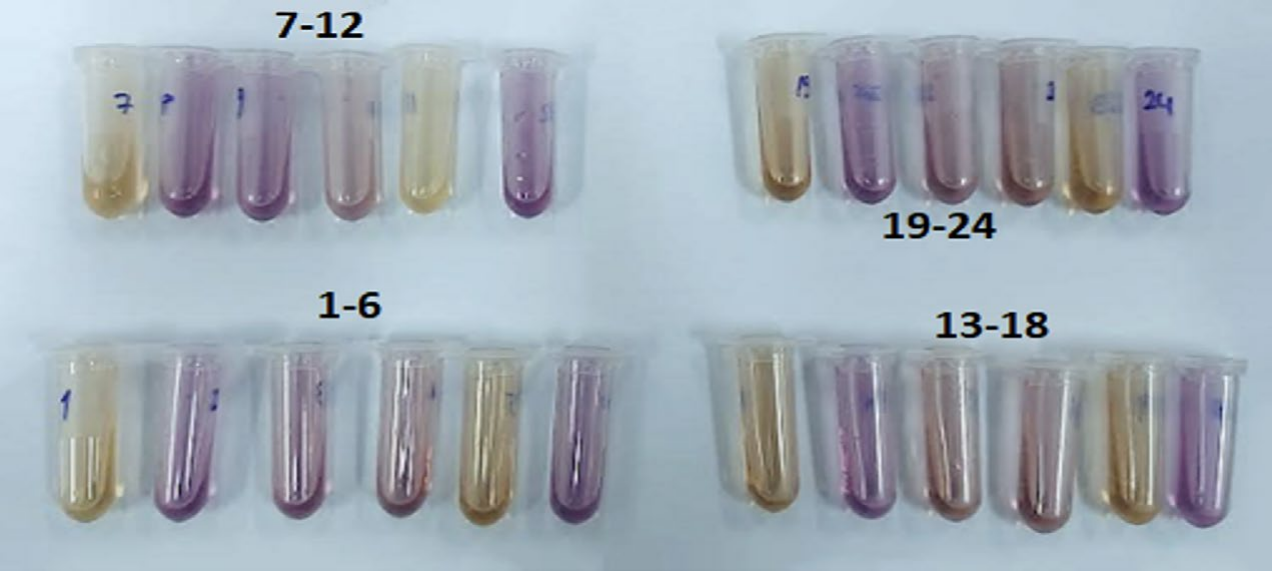
Representative images illustrating the DPPH radical scavenging reaction. The color transition from purple to yellow indicates increasing antioxidant activity of the tested artificial saliva formulations at different sample volume.

The assay was performed according to the method described by Brand-Williams et al.^7^ Briefly, aliquots of 50, 100, and 200 µl of each sample solution were mixed with 1.0 ml of a 0.1 mM DPPH solution prepared in methanol. Following vigorous vortexing, the reaction mixtures were incubated in the dark at room temperature for 30 min. Absorbance was then measured at 517 nm. Methanol containing DPPH solution without sample served as the control, while methanol alone was used as the blank. All measurements were performed in triplicate. Radical scavenging activity was calculated as percentage inhibition using the following formula:

% Inhibition = [(CA − AO) / CA] × 100,

where CA represents the absorbance of the control and AO represents the absorbance of the sample. Lower absorbance values at 517 nm indicated higher free-radical scavenging activity (Fig 6).

### Total Phenolic Compound Determination 

**Fig 7:**
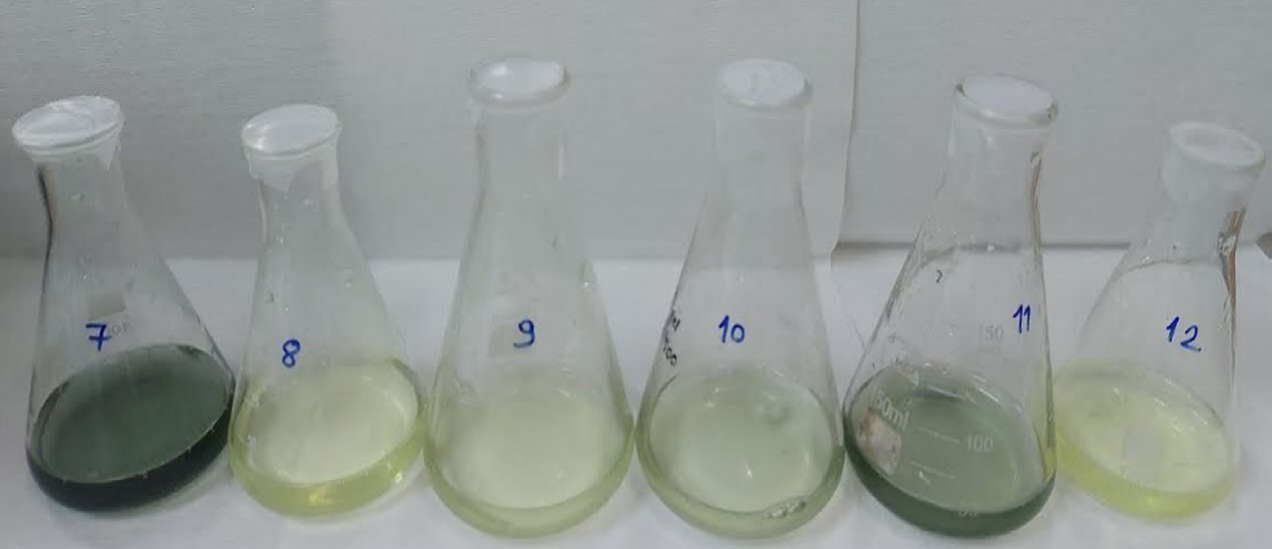
Representative visual appearance of artificial saliva samples during total phenolic content determination using the Folin–Ciocalteu method. Variations in color intensity qualitatively reflect differences in phenolic compound concentration among the tested formulations prior to spectrophotometric quantification.

**Fig 8:**
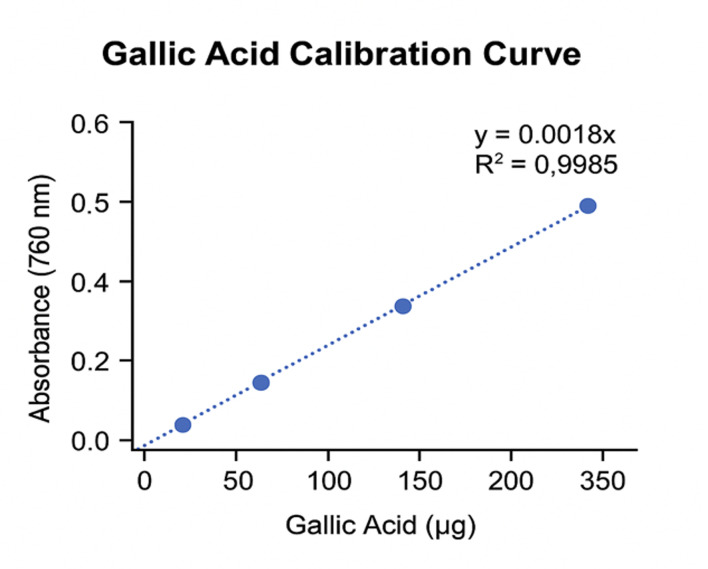
Gallic acid calibration curve used for the quantification of total phenolic content, expressed as gallic acid equivalents (mg GAE/100 ml).

Total phenolic content was determined according to the method described by Singleton.^31^ This analysis was performed to provide a chemical correlate for the observed antioxidant activity, as phenolic compounds are widely recognized as major contributors to radical scavenging capacity in plant-derived extracts (Fig 7). Briefly, 1.0 ml of each sample solution was transferred into 100-ml Erlenmeyer flasks, followed by the addition of 24.0 ml of distilled water. Subsequently, 0.5 ml of Folin–Ciocalteu reagent was added, and after 3 min, 1.5 ml of 2% Na₂CO₃ solution was introduced. The mixtures were shaken at 120 rpm for 2 h at room temperature in the dark. Absorbance was measured at 760 nm against distilled water, and total phenolic content was calculated as gallic acid equivalents (mg GAE/100 ml) using a gallic acid calibration curve (Fig 8).

### Evaluation of Antimicrobial Activity

The antimicrobial activity of artificial saliva formulations was evaluated using a disk-diffusion assay. Lyophilized reference strains of *Streptococcus mutans* (ATCC 25175) and *Staphylococcus aureus* (ATCC 29213) were revived according to the manufacturer’s instructions and cultured on chocolate agar (AEM Medical; Ankara, Türkiye) and 5% sheep blood agar (Spesera; Istanbul, Türkiye), respectively. A clinical isolate of *Candida albicans* was identified by MALDI-TOF MS and subcultured on Sabouraud dextrose agar (Biocell; Istanbul, Türkiye).

Microbial suspensions were adjusted to a 0.5 McFarland standard and evenly inoculated onto agar surfaces using sterile cotton swabs. Sterile 5-mm paper disks impregnated with 10 µl of each artificial saliva formulation were placed onto the inoculated plates. A total of 24 formulations were tested for each microorganism. Plates were incubated at 37°C and evaluated after 24 h for inhibition-zone formation. As no inhibition zones were observed, further readings at 48 and 72 h were not performed.

## RESULTS

### Viscosity Measurement Findings

The viscosity values obtained for all experimental groups are presented in Table 1. All measurements were performed in triplicate (n = 3) and are expressed as mean ± standard deviation. Statistical analysis was carried out using SPSS software (SPSS; Chicago, IL, USA). One-way ANOVA revealed statistically significant differences among the groups (p < 0.05). Post-hoc comparisons were performed using Duncan’s multiple range test to identify differences between individual formulations.

**Table 1:** Viscosity values presented as mean ± SD

Matrix	Formulation	Value (mean ± SD)
Low CMC	Control	70.3 ± 0.1^e^
	*Cynara cardunculus*	56.6 ± 0.3^d^
	*Origanum vulgare*	44.0 ± 4.1^b^
	*Equisetum arvense*	35.1 ± 0.1^a^
	*Hypericum perforatum*	39.4 ± 2.7^ab^
	*Olea europaea*	51.0 ± 0.7^c^
High CMC	Control	39.9 ± 2.2^a^
	*Cynara cardunculus*	39.8 ± 2.2^a^
	*Origanum vulgare*	61.6 ± 1.0^c^
	*Equisetum arvense*	53.2 ± 2.2^b^
	*Hypericum perforatum*	51.2 ± 3.2^b^
	*Olea europaea*	66.9 ± 2.1^c^
Low mucin	Control	56.8 ± 2.1^a^
	*Cynara cardunculus*	59.0 ± 1.3^a^
	*Origanum vulgare*	72.9 ± 0.4^b^
	*Equisetum arvense*	71.6 ± 1.2^b^
	*Hypericum perforatum*	62.9 ± 4.7^a^
	*Olea europaea*	70.0 ± 3.8^b^
High mucin	Control	62.8 ± 0.7^ab^
	*Cynara cardunculus*	61.3 ± 0.6^a^
	*Origanum vulgare*	86.0 ± 1.8^d^
	*Equisetum arvense*	67.1 ± 1.1^c^
	*Hypericum perforatum*	62.0 ± 1.0^a^
	*Olea europaea*	66.5 ± 2.8^bc^
Within the same group, values with different superscript letters are statistically different (p<0.05).		

### Cytotoxicity Evaluation on Gingival Fibroblasts

**Fig 9:**
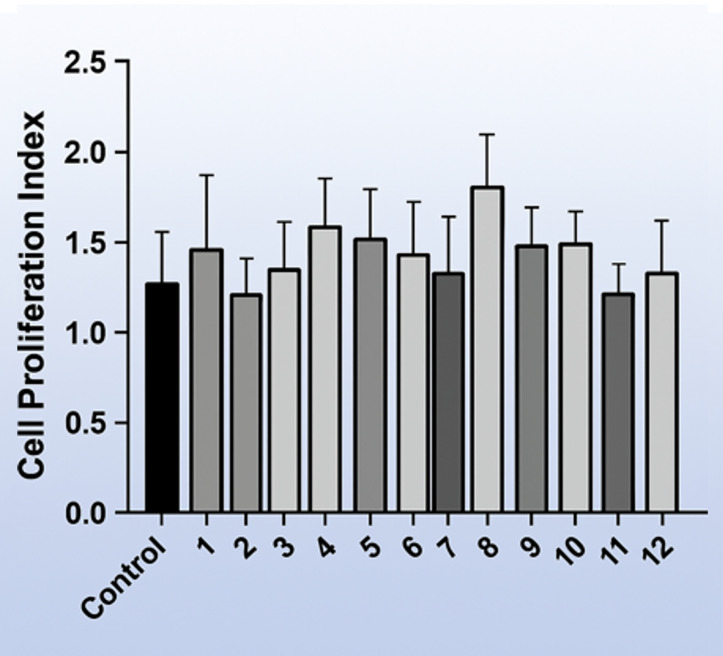
Cell viability of gingival fibroblasts after 24-h exposure to artificial saliva formulations, assessed by the MTT assay. Data are presented as mean ± standard deviation (n = 3) (product numbers 1-12).

**Fig 10:**
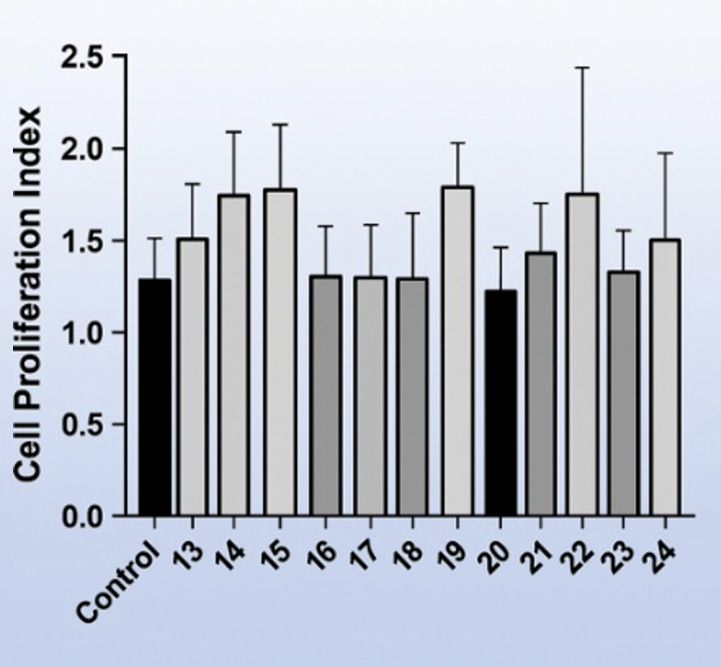
Cell viability of gingival fibroblasts after 24-exposure to artificial saliva formulations, assessed by the MTT assay. Data are presented as mean ± standard deviation (n = 3) (product numbers 13-24).

The cytotoxic effects of the artificial saliva formulations were evaluated using the MTT assay on gingival fibroblast cells at the fourth passage. Cell viability results are shown in Figs 9 and 10. When compared with untreated control cells cultured in standard growth medium, none of the experimental formulations resulted in a statistically significant reduction in cell viability after 24 h of exposure (p > 0.05). Extract-free artificial saliva formulations served as formulation controls and exhibited comparable cell viability to the negative control group.

### Antioxidant Activity Evaluation (DPPH Assay)

**Fig 11:**
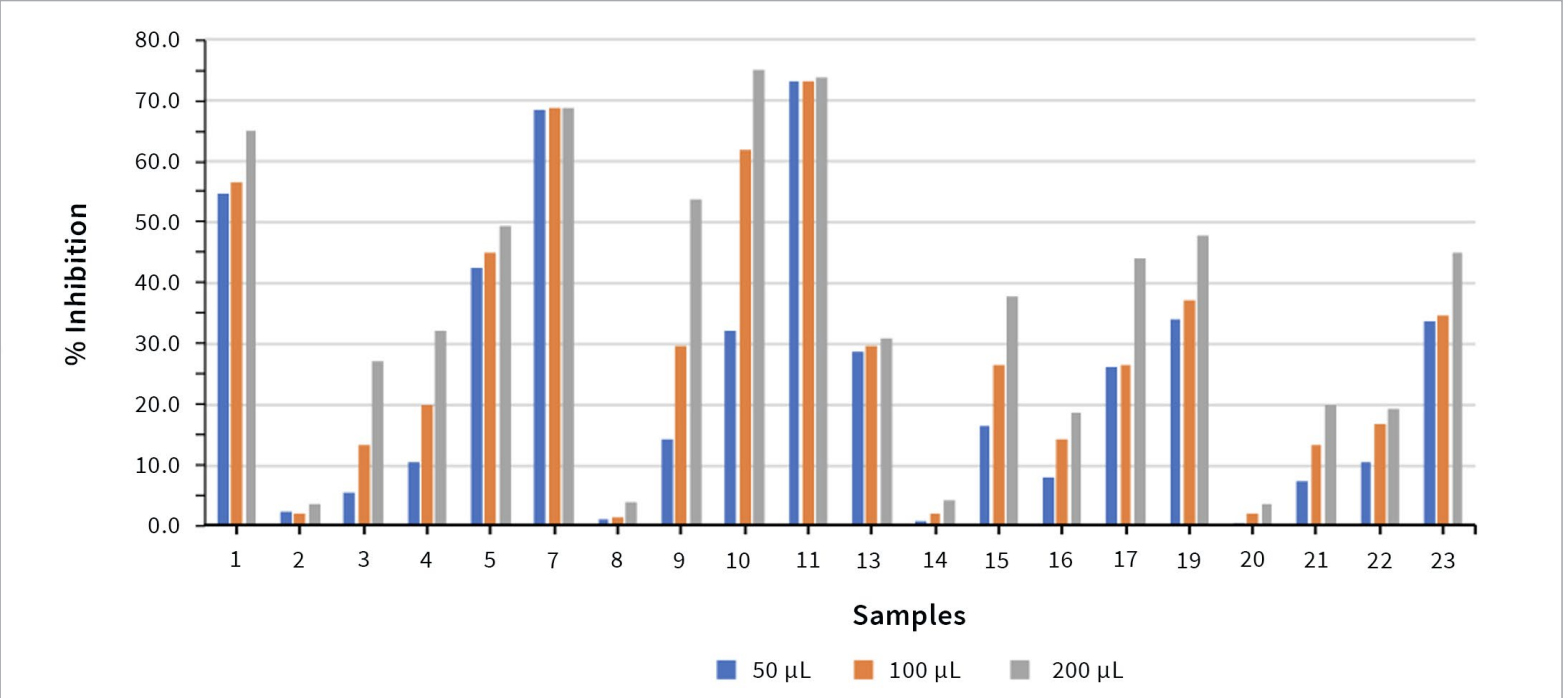
DPPH radical scavenging activity of artificial saliva formulations containing different plant extracts. Data are presented as percentage inhibition, with extract-free formulations serving as controls. A dose-dependent increase in antioxidant activity was observed.

The antioxidant activity of the artificial saliva formulations was assessed using the DPPH radical scavenging assay. Quantitative results are presented in Fig 11. Formulations containing plant extracts demonstrated statistically significantly higher radical scavenging activity compared with extract-free control formulations (p < 0.05). Among the tested extracts, formulations containing *Olea europaea* and *Hypericum perforatum* exhibited the highest antioxidant activity. A dose-dependent increase in radical scavenging activity was observed with increasing formulation volume.

### Antimicrobial Activity Results

No antimicrobial activity was observed for any of the tested artificial saliva formulations against *Streptococcus mutans, Staphylococcus aureus*, or *Candida albicans*. At the 24-h evaluation, complete microbial growth was observed around all disks, and no inhibition zones were detected. As no antimicrobial effect was evident at the initial time point, extended incubation at 48 and 72 h was deemed unnecessary.

## DISCUSSION

The present study investigated the physicochemical properties, cytocompatibility, and antioxidant activity of an artificial saliva formulation incorporating selected plant extracts. Within the limitations of an in-vitro experimental design, as an exploratory, formulation-level in-vitro investigation. The formulations exhibited viscosity values within the reported physiological range of natural saliva, showed no detectable cytotoxic effects on gingival fibroblast cells, and demonstrated measurable antioxidant activity, particularly in formulations containing *Olea europaea* and *Hypericum perforatum* extracts.

Artificial saliva formulations have long been investigated for their ability to replicate the physicochemical and protective functions of natural saliva. Previous studies have emphasized that viscosity plays a central role in maintaining oral lubrication and mucosal protection.^4,30^ The viscosities obtained in the present study (19–35 µPa) fall within the range reported for resting and stimulated human saliva and are consistent with earlier findings by Ionta et al,^17^ who demonstrated that formulations within this range can provide adequate lubrication and film formation. In the present study, viscosity was assessed as a relative parameter to enable controlled comparison between formulations rather than absolute rheological characterization, which limits direct quantitative comparison with all published rheological datasets. The use of mucin and sodium carboxymethyl cellulose (CMC) as base matrices reflects common approaches in saliva substitute research, with mucin-based formulations reported to more closely reproduce the viscoelastic and boundary-lubricating behavior of natural saliva compared with polymer-based systems.^19^

In cell culture studies, MTT assays performed on gingival fibroblasts demonstrated that the tested artificial saliva formulations were non-cytotoxic and exhibited good biocompatibility. Qualitative microscopic observations of cell morphology supported these findings. Based on its in-vitro antioxidant activity and cytocompatibility profile, the developed formulation may serve as a promising biomimetic saliva substitute candidate. However, further in-vivo and clinical studies are required to evaluate its clinical performance, safety, and potential advantages compared to existing

The cytotoxicity evaluations performed on gingival fibroblast cells indicated that the tested artificial saliva formulations did not induce detectable cytotoxic effects under the applied in-vitro conditions. MTT assay results showed that none of the experimental groups resulted in a statistically significant reduction in cell viability compared with control cultures. These observations are in agreement with previous in-vitro studies, such as that of Manosroi et al,^21^ who reported preserved fibroblast viability in artificial saliva formulations enriched with *Basella alba* mucilage, and Tokur and Aksoy,^34^ who demonstrated that herbal components, when used within appropriate concentration ranges, do not adversely affect fibroblast survival.

Collectively, these findings support the cytocompatibility of the tested formulations at the cellular level; however, this observation is limited to short-term in-vitro exposure conditions and should not be extrapolated to in-vivo safety or clinical performance. While the results suggest that plant-based artificial saliva formulations can be designed without inducing fibroblast toxicity, further in-vivo and clinical investigations are required to determine their biological performance, safety, and potential relevance under physiological conditions.

Antioxidant analyses showed that formulations containing *Olea europaea* and *Hypericum perforatum* extracts exhibited higher radical scavenging activity in the DPPH assay compared with extract-free controls. This observation is consistent with previous reports describing the high phenolic and flavonoid content of these plant species and their associated in-vitro antioxidant properties.^5,22^ The concordance between the present results and earlier studies supports the chemical plausibility of these extracts contributing to the observed radical scavenging activity within the formulation.

In the context of artificial saliva, such antioxidant activity may be relevant for counteracting oxidative stress under experimental conditions; it is important to bear in mind that the present findings are limited to a chemical assay system and do not demonstrate biological antioxidant effects at the tissue or clinical level. Therefore, no direct conclusions can be drawn regarding biological efficacy, tissue healing, or clinical benefit. Further in-vivo and clinical studies would be required to clarify the potential relevance of these findings for xerostomia management, particularly in patient populations exposed to increased oxidative stress.^32^

In addition to antioxidant activity, the antimicrobial potential of the final artificial saliva formulations was directly evaluated using standard disk diffusion assays against *Streptococcus mutans, Staphylococcus aureus, *and* Candida albicans*. Although several of the selected plant extracts have been reported to exhibit antimicrobial properties in isolated extract studies, no antimicrobial activity was detected in the present formulation under the tested conditions. This finding suggests that the concentrations used, the aqueous formulation matrix, or interactions with salivary components may have limited the availability or efficacy of antimicrobial compounds. These results highlight the importance of evaluating antimicrobial performance at the final formulation level rather than inferring activity from extract-level data alone. Future studies may explore alternative concentrations, delivery systems, or complementary antimicrobial agents if antimicrobial functionality is a targeted outcome.

A distinctive aspect of this study is the ethnobotanical rationale guiding the selection of plant extracts. Extracts derived from *Cynara cardunculus* (artichoke), *Origanum vulgare* (thyme), *Equisetum arvense* (horsetail), *Olea europaea* (olive leaf), and *Hypericum perforatum* (St. John’s Wort)—all native to the Aegean region—were selected based on consistent literature evidence supporting their antioxidant potential and suitability for aqueous formulations.^25^ Despite their documented bioactive profiles, these plant extracts have rarely been explored within the context of artificial saliva formulations or dental biomaterials.

The use of regionally sourced botanical materials aligns with broader trends toward sustainable and biocompatible biomedical products.^3^ Nevertheless, the present study was intentionally designed as an exploratory in-vitro investigation and does not aim to establish clinical superiority or equivalence to commercially available saliva substitutes. Key aspects required for clinical translation – including sensory characteristics, standardized long-term stability, and direct comparison with existing commercial formulations – were therefore beyond the scope of this work. Accordingly, the findings should be interpreted as preliminary and hypothesis-generating, providing a formulation-level foundation for future in-vitro, in-vivo, and comparative studies rather than confirmatory clinical evidence.

## CONCLUSION

In this study, an artificial saliva formulation incorporating selected plant extracts was developed and evaluated using in-vitro physicochemical, cytocompatibility, and antioxidant assays. The formulations exhibited viscosities within the physiological range of natural saliva and did not induce cytotoxic effects in gingival fibroblast cultures under the experimental conditions applied. In addition, plant-extract–containing formulations demonstrated measurable antioxidant activity in-vitro.

While these findings indicate that the proposed formulation possesses favorable in-vitro characteristics, they are limited by the scope of the experimental design. Further studies, including in-vivo models, comparative evaluations with commercial products, assessment of sensory properties, and long-term stability are necessary before any clinical relevance can be inferred. The present findings provide a formulation-level foundation that may support further optimization and evaluation of artificial saliva systems in future studies.

## ACKNOWLEDGMENTS

The authors gratefully acknowledge the financial and infrastructural support provided by the Dokuz Eylül University Scientific Research Projects Coordination Unit (BAP) for this study. This research was conducted within the framework of a BAP project aimed at promoting innovative and locally developed biomedical materials. The authors would like to extend their sincere appreciation to the Faculty of Dentistry, Dokuz Eylül University, for providing laboratory facilities and technical assistance throughout the experimental procedures. Special thanks are also due to all members of the research team who contributed to the formulation development, physicochemical analyses, and in-vitro evaluations. Their valuable insights and collaborative efforts were instrumental in the successful completion of this study. The continuous encouragement and academic guidance offered by the university’s research community have been essential in achieving the objectives of this project.

## REFERENCES
